# The Opening of High-Speed Railways, the Improvement of Factor Allocation Efficiency between Regions, and the City’s Environmental Quality Improvement

**DOI:** 10.3390/ijerph20054648

**Published:** 2023-03-06

**Authors:** Bochao Zhang, Wanhao Dong, Jin Yao

**Affiliations:** 1 Institute of Economics, Shanghai Academy of Social Sciences, Shanghai 200020, China; 2 School of Public Finance and Administration, Shanghai Lixin University of Accounting and Finance, Shanghai 201620, China

**Keywords:** high-speed railway opening, allocative efficiency of factors, environmental pollution, technological innovation, urban heterogeneity, PSM-DID

## Abstract

Based on the panel data of prefecture-level cities in China from 2006 to 2019, this paper uses the PSM-DID method to empirically test the internal impact mechanism among high-speed railway opening, inter-regional factor allocation efficiency, and urban environmental governance. The research results show that: (1) There is a serious factor-misallocation problem among prefecture-level cities in China. From 2006 to 2019, the factor misallocation between prefecture-level cities led to an average annual loss of total factor productivity in China’s economy of 52.5%, an average labor misallocation of 23.16%, and an average capital misallocation of 18.69%. Since 2013, capital misallocation has exceeded labor misallocation as the main reason for factor misallocation among prefecture-level cities in China. (2) The opening of high-speed railways can promote the efficiency of urban factor allocation through the technological innovation effect, the foreign investment attraction effect, and the population agglomeration effect. The improvement of urban factor allocation efficiency can promote the improvement of urban environmental quality through the effects of industrial structure optimization, income enhancement, and human capital agglomeration. Therefore, the opening of a high-speed railway can improve urban environmental quality through the intermediary effect of improving the efficiency of urban factor allocation; that is, the opening of a high-speed railway has a dual positive effect of economic efficiency and environmental quality improvement. (3) The optimization effect of factor allocation and the environmental governance effect of the opening of high-speed railways have strong urban scale heterogeneity, urban characteristic heterogeneity, and regional heterogeneity. The research content of this paper has important guiding significance for the construction of China’s new development paradigm, accelerating the construction of “a unified national market,” and green and low-carbon development.

## 1. Introduction

Affected by multiple factors, such as the continuous and repeated COVID-19 pandemic and the complex and changeable international environment, China’s economic development is facing the triple pressure of demand contraction, supply shock, and weakening expectations. Accelerating the free and efficient flow of various domestic factor resources and improving the allocation efficiency, stimulating the vitality of various factors, and then improving the overall total factor productivity and economic development vitality are the inevitable requirements for dealing with the triple pressure. To promote the free and efficient flow and allocation of factors, on the one hand, it is necessary to eliminate the dual economic structure [1], the household registration system [2], industry monopolies [3], ownership discrimination [4], market segmentation [5], and other institutional obstacles to the flow and allocation of factor resources.

On the other hand, it is necessary to optimize the hardware environment for factor flow and efficient allocation by optimizing infrastructure such as transportation networks [6,7]. In the current context of insufficient domestic demand, China will inevitably need to increase investment in new infrastructure construction, such as high-speed railways, in the future. On the one hand, it can improve domestic infrastructure; on the other hand, it can increase domestic investment demand to drive economic growth. What’s more, the continuous improvement of the high-speed railway network will inevitably have an important impact on the way and efficiency of China’s factor flow and allocation and will have a significant impact on the construction of a domestic economic cycle support system. Therefore, in this context, it is of great practical significance to study the economic effect of high-speed rail from the perspective of resource allocation.

The imbalance between economic development and ecological protection has become an important aspect of the main contradiction in Chinese society, which must be paid great attention to and find an effective path to solve it, which means that China’s economic development will face increasingly tight environmental regulatory constraints in the future. Therefore, when studying the impact of high-speed rail construction on the economy and society, we cannot ignore its environmental effects. Existing studies have shown that the opening of high-speed railways has significant direct environmental effects, mainly reflected in the fact that high-speed railways, as a new mode of travel with high speed, punctuality, safety, and comfort, can significantly change the overall structure of travel modes for members of society and can produce alternative effects on other means of transportation [8].

In addition, high-speed rail can also indirectly have a significant impact on environmental pollution through its economic effect as an intermediary mechanism [9]. This leads to the following questions worthy of in-depth exploration: What is the intrinsic relationship between the opening of high-speed railways, factor allocation efficiency, and environmental pollution? While high-speed rail significantly affects the efficiency of factor allocation in China, can it also be used as an intermediary mechanism to affect environmental quality? Through the in-depth discussion of the above issues, it is conducive to clarifying the intrinsic relationship and deep-seated impact mechanism between the resource allocation effect and the environmental effect of high-speed railway, and then more comprehensively understanding whether high-speed railway can help achieve the win-win effect of improving resource allocation efficiency and improving environmental quality in line with the requirements of the current era.

The content of this paper is structured as follows: The second part is a literature review; the third part is a theoretical analysis and research hypothesis; the fourth part is an empirical strategy; the fifth part is the empirical results and analysis; and the sixth part is a heterogeneity analysis; the seventh part is the main conclusions and countermeasures.

## 2. Literature Review

The existing literature related to the research theme of this paper mainly includes two aspects: one is the research on the resource allocation effect of transportation infrastructure such as high-speed railways, and the other is the research on the environmental effect of transportation infrastructure such as high-speed railways. Among them, the research on the resource allocation effect of high-speed railways and other transportation infrastructure is mainly carried out from the following aspects: First, the resource allocation effect between enterprises of high-speed railway and other transportation infrastructure is examined from a micro perspective. In terms of theoretical analysis, Liu and Hu [10] believe that the construction of transportation infrastructure, such as high-speed railways, can break the market segmentation caused by local protectionism and improve the efficiency of factor allocation between enterprises by promoting reasonable, orderly, and full competition among enterprises. Zhou et al. [11] and Li et al. [12] believe that the opening of high-speed railways can improve the accuracy of the division of labor between enterprises, expand the scope of market space, and accelerate the level of economic agglomeration to improve resource misallocation between enterprises. At the level of empirical analysis, most of the above studies match the database data of Chinese industrial enterprises with the data of high-speed railway or other transportation infrastructure opening data and the data at the city and provincial levels. For example, Li et al. [13] used the database data of Chinese industrial enterprises from 1998 to 2013 to match the data of “four horizontal and four vertical” high-speed railway opening stations and found that the opening of high-speed railway can significantly improve the capital misallocation between enterprises, but the distortion effect on the product market is not significant, and the factor allocation effect has heterogeneity of ownership. Bu et al. [6] used the 2001–2007 Chinese industrial enterprise database to empirically find that highway construction can effectively correct the labor misallocation and intermediate input misallocation between enterprises, which led to an increase in total output of 9.8% and 4.39%, respectively. Zhang [14] used the database of Chinese industrial enterprises from 2009 to 2013 and found that the opening of high-speed railways can effectively reduce the misallocation of production factors between enterprises, and the misallocation improvement effect has industry heterogeneity and city-scale heterogeneity. Li et al. [12] also used the 1998–2007 Chinese industrial enterprise database to find that the opening of expressways had a significant improvement effect on the misallocation of factors among manufacturing firms in non-central cities. In summary, the theoretical and empirical research conclusions at the micro level are basically the same, and the existing studies show that the construction of transportation infrastructure, such as high-speed railways, can significantly improve the factor misallocation between enterprises. The second is to examine the resource allocation effect of high-speed railway and other transportation infrastructure construction at the city and provincial levels. Li et al. [15] used 2000–2016 Chinese prefecture-level city data to find that the opening of high-speed railways can accelerate labor circulation and capital accumulation, optimize the efficiency of urban resource allocation, and promote the upgrading of urban industrial structure. Wei and Zhang [16] used provincial panel data to find that the opening of high-speed railways can alleviate the problem of overcapacity by reducing the misallocation of capital and labor. Niu and Cui [17] used prefecture-level city panel data to empirically find that the opening of high-speed railways can improve labor misallocation by promoting labor mobility and industrial agglomeration, and that its improvement effect has significant geographical circle characteristics and spatial spillover effects. On the basis of summarizing the shortcomings of previous research, Wen et al. [7] used a 1998–2013 database of Chinese industrial enterprises, matched traffic data and geographic vector data, added up the misallocation of resources between enterprises into urban resource misallocation, and comprehensively investigated the resource allocation effects of highways, ordinary railways, civil aviation, and high-speed railways on the basis of prefecture-level city panel data. The results showed that the improvement effect of high-speed railway on urban resource allocation is about 3.1%, the distortion effect of expressway on urban resource allocation is as high as 2.14%, and the role of ordinary railway and civil aviation is not significant or stable.

In addition to studying the resource allocation effect of high-speed rail, other scholars focused on the environmental effects of high-speed rail and other transportation infrastructure. According to existing studies, the environmental effects of high-speed rail are mainly divided into direct and indirect effects. The direct effects are mainly reflected in the fact that high-speed rail, as a new type of transportation mode, has alternative effects on other traditional transportation modes (highway, general railway) by virtue of its high efficiency, comfort, environmental protection, and energy conservation, and thus achieves the effects of energy conservation, emission reduction, and environmental improvement [8]. High-speed rail has greatly changed people’s daily travel choices, resulting in the traffic substitution effect or Mohring effect [18], optimizing the overall transport structure of the region, and reducing the level of environmental pollution. In addition, some scholars have analyzed the positive role of the substitution effect of high-speed railways on climate change from the perspective of the life cycle. For example, Chester and Horvath [19] evaluated the impact of a high-speed railway system on environmental pollution based on the California corridor and found that the substitution effect of high-speed railways on road travel will improve exhaust emissions when considering the impact of emerging vehicles, infrastructure, and the supply chain life cycle. At the same time, Zhang and Feng [20] believed that despite the effective replacement of traditional travel modes, high-speed rail has the effect of creating travel demand, which can increase energy consumption and pollutant emissions by inducing new travel demand. In addition, it can be seen from this that the final effect of the direct environmental effect of high-speed rail depends on the priority of its substitution effect and travel demand creation effect. In terms of the indirect effect of high-speed rail on environmental governance, as high-speed rail mainly focuses on passenger transport, it has a more important impact on the service industry with its strong mobility of production factors and promotes the spatial agglomeration and development of the service industry. The agglomeration and development of the service industry is an important link in the adjustment and upgrading of the industrial structure and has a significant emission reduction effect [21]. Faber [22] further pointed out that the high-speed railway will make the service industry gather in the central cities where the high-speed railway is open and have a restraining effect on the development of the service industry in the surrounding small and medium-sized cities. This “polarization effect” of the service industry agglomeration development will lead to the spatial spillover effect of pollutants.

In summary, in terms of the resource allocation effect of high-speed railway openings, whether at the micro level or at the macro level, existing studies generally agree that the opening of high-speed railways has a significant resource allocation effect, which can significantly alleviate the misallocation of resources between enterprises or between cities. However, the existing studies have not further extended it to the field of urban environmental pollution to explore the impact mechanisms of changes in resource allocation efficiency caused by the opening of high-speed railways on urban environmental pollution. In terms of the research on the environmental effects of high-speed railway opening, when analyzing the mediating mechanism of a high-speed railway opening affecting urban environmental governance, few studies examine the internal impact mechanism of the environmental effect of high-speed railway opening from the perspective of resource allocation. Based on the above research status, the possible marginal contributions of this paper are as follows: Firstly, this paper constructs a quantitative evaluation model of factor allocation efficiency to quantitatively evaluate the factor misallocation level between prefecture-level cities in China. Secondly, this paper examines the internal mechanism of the opening of a high-speed railway’s impact on urban environmental quality from the perspective of inter-regional factor allocation efficiency. Internal intermediary mechanisms of the opening of a high-speed railway affecting the inter-regional factor allocation efficiency and the internal mechanism by which the change in factor allocation efficiency affects the quality of the urban environment have been explored. Thirdly, this paper examines the impact and internal mechanisms of high-speed railways on the efficiency of inter-regional factor allocation and urban environmental quality and examines the economic and social effects of high-speed railway openings from the dual perspectives of “economic efficiency” and “environmental quality improvement”, which can put forward more comprehensive evaluation criteria for the economic and social effects of high-speed railways. Fourthly, this paper examines the internal relationship between the opening of high-speed railways, the efficiency of factor allocation, and urban environmental pollution from the multidimensional perspective of urban scale heterogeneity, urban characteristics heterogeneity, and regional heterogeneity, which has important reference significance for China to formulate more accurate and scientific high-speed railway planning in the future.

## 3. Theoretical Analysis and Research Hypotheses

### 3.1. Analysis of the Impact Mechanism of High-Speed Railway Opening on the Efficiency of Urban Factor Allocation

Numerous studies have shown that the opening of high-speed railways has a significant effect on improving the efficiency of resource allocation [7,16,23]. From the perspective of its influence mechanism, most existing studies believe that the opening of high-speed railways can improve the accessibility of cities [14], weaken China’s labor market segmentation, promote a freer and more efficient flow of labor and capital factors [17], and then achieve the effect of improving the efficiency of resource allocation. In addition to the above influence mechanism, this paper will continue to further explore the internal mechanism of high-speed railway opening and how it can significantly improve factor allocation efficiency (especially inter-city factor allocation efficiency) from the capital side, labor side, and technology side. There are three paths—the technical innovation effect, the foreign investment attraction effect, and the population agglomeration effect—to promote the improvement of the efficiency of production factor allocation between cities.

Technological innovation effect. The opening of high-speed railways can correct the misallocation of resources between cities and improve the efficiency of resource allocation between cities by promoting regional technological innovation. Because of its significant advantages of punctuality, high speed, and convenience, high-speed rail can become the first choice for high-quality talents who are highly sensitive to time requirements, from which it can be inferred that the opening of high-speed rail can promote the more efficient and frequent flow of high-quality talents between regions. Because high-quality talents are an important carrier of knowledge flow and technological innovation [24], this is bound to further lead to the spread and spillover of knowledge and technical elements between regions, which in turn is conducive to consolidating the innovation resource endowment of high-speed railway opening cities and surrounding related cities and stimulating the vitality of urban technological innovation. The improvement of technological innovation vitality is conducive to breaking the original regional competition pattern, triggering the comparative advantages of different cities’ industrial economies, and promoting the level of market competition between regions [25]. The “survival of the fittest” effect brought about by competition can promote the withdrawal of inefficient enterprises from the market, release the flow of production factors to high-efficiency enterprises [26], and ultimately optimize the efficiency of factor allocation.

Foreign investment attraction effect. The improvement of infrastructure, such as high-speed railways, can achieve the effect of attracting foreign investment by significantly improving the economic operation environment of cities along the route [16,27], and this effect is long-standing [28]. The opening of a high-speed railway can significantly improve the popularity and attractiveness of a city and enhance its level of opening to the outside world. On the one hand, foreign-invested enterprises can use transnational production and sales networks to achieve economies of scale and enhance their competitiveness, and at the same time they will exert competitive pressure on enterprises in the host country, promote local market competition, and then force local enterprises to optimize their own resource allocation efficiency. On the other hand, the orientation of FDI will effectively alleviate the imbalance in capital market allocation in China [29], that is, FDI tends to choose private enterprises with high operating efficiency and more serious financing constraints as investment objects to effectively correct the imbalance in the allocation of capital factors.

Population agglomeration effect. According to existing research, the opening of high-speed railways can accelerate population agglomeration in cities along the high-speed railway line and achieve the most optimal population allocation [30]. On the one hand, the spatial agglomeration represented by the population will improve the level of local urbanization, accelerate the urbanization of agricultural transfer populations, promote the equalization of basic public services, eliminate obstacles to cross-regional allocation of labor, and then increase the number of effective laborers in cities, increase the matching opportunities between enterprises and labor, and improve the employment probability and spatial allocation efficiency of labor [31]. On the other hand, population agglomeration is also conducive to further driving industrial agglomeration, and the improvement of industrial agglomeration level is conducive to giving full play to financial externalities to alleviate the misallocation of credit resources [32] and then effectively disposing of zombie enterprises, releasing the capital and labor factors that were originally in an inefficient allocation state to flow to high-efficiency enterprises, improving the dynamic replacement efficiency of market entities, and ultimately achieving the effect of improving the efficiency of resource allocation. In addition, the improvement of industrial agglomeration level can also guide capital flow to high-quality enterprises by lowering the entry threshold of enterprises [33] and ultimately alleviate capital misallocation.

Based on the above analysis, the first hypothesis to be tested in this paper is proposed here.

**Hypothesis** **1:** *The opening of high-speed railways can promote the improvement of urban factor allocation efficiency through the technological innovation effect, the foreign investment attraction effect, and the population agglomeration effect.*

### 3.2. Analysis of the Influence Mechanism of Urban Factor Allocation Efficiency on Urban Environmental Quality

This paper mainly attempts to explain that the opening of high-speed railways can improve the quality of urban environments by improving the efficiency of urban factor allocation, i.e., the improvement of urban factor allocation efficiency is an important intermediary mechanism for the opening of high-speed railways to affect the quality of urban environments. Therefore, this section will focus on the mechanisms through which the improvement of urban factor allocation efficiency mainly affects the environmental quality of cities. The mechanism of improving the efficiency of urban factor allocation and affecting the quality of the urban environment mainly lies in:

Industrial structure optimization effect. The improvement of urban factor allocation efficiency can reduce urban environmental pollution by promoting the optimization and upgrading of urban industrial structures. The multi-dimensional factor misallocation in China is one of the main obstacles restricting the optimization and upgrading of China’s industrial structure. According to the research of Bai and Zhang [34], according to the current status of industrial development in China, correcting the misallocation of factors between industries can shrink the scale of traditional industries with high pollution and high energy consumption and expand the scale of emerging industries such as technology-intensive industries, thereby achieving the effect of optimizing the industrial structure. Zhang et al. [35] found that correcting the misallocation of factors between different factor-intensive industries can also significantly increase the scale and proportion of technology-intensive industries and then promote the optimization and upgrading of industrial structure. It can be seen that the optimization and upgrading of industrial structure mean that the scale and proportion of high-pollution, high-energy-consuming industries shrink and the scale of technology-intensive industries expand. The optimization and upgrading of industrial structures will inevitably reduce the environmental pollution generated by urban economic activities and then improve the quality of the urban environment. Therefore, the improvement of urban factor allocation efficiency can simultaneously improve the quality of the urban environment through the effect of industrial structure optimization.

Revenue-boosting effects. The improvement of urban factor allocation efficiency means an increase in total factor productivity and total output of the urban economy, which in turn promotes urban economic growth. On the one hand, urban economic growth increases government fiscal revenue so that the local government can have more abundant financial resources to invest in environmental pollution control. On the other hand, urban economic growth makes urban residents’ income increase rapidly, and the increase in urban residents’ income will make them pay more attention to urban environmental quality, which in turn will force the government to increase environmental regulation and reduce the emission of environmental pollutants [30]. Therefore, the improvement of urban factor allocation efficiency can strengthen the government’s environmental regulation intensity and optimize urban environmental quality through the income increase effect.

Human capital agglomeration effect. As mentioned above, high-speed rail has become the first choice for high-quality talents to travel because of its punctual and high-speed characteristics, and high-quality talents are an important carrier of human capital. Therefore, the flow and allocation of human capital caused by the opening of high-speed railways will not only optimize the efficiency of urban factor allocation but also trigger the effect of human capital agglomeration; that is, the opening of high-speed railways can promote the flow and the allocation efficiency of human capital through high efficiency and improve the concentration of human capital in cities where high-speed railways are opened. On the one hand, this is conducive to updating the knowledge and technology of urban enterprises in cities with the opening of high-speed railways and to the wide application of energy-saving and environmental protection technologies. On the other hand, highly concentrated human capital also provides an intellectual foundation for cities to strengthen environmental governance, which can provide more optimal policy combination options for the government to improve environmental quality. Highly concentrated human capital is also conducive to the government’s efforts to improve the implementation effect of administrative orders and market incentives for environmental regulation policies so as to achieve the purpose of optimizing environmental quality.

**Figure 1:**
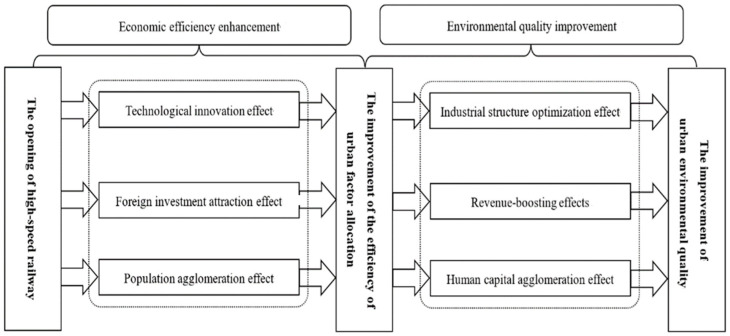
Schematic diagram of the relationship between the opening of high-speed railways, resource allocation efficiency, and urban environmental pollution.

The relationship among the opening of high-speed railway, the efficiency of inter-regional factor allocation and environmental quality is shown in Figure 1. Based on the above analysis, the second and third hypotheses to be tested in this paper are proposed here.

**Hypothesis** **2:** *The improvement of urban factor allocation efficiency can promote the improvement of urban environmental quality through an industrial structure optimization effect, revenue-boosting effects, and a human capital agglomeration effect.*

**Hypothesis** **3:** *The opening of high-speed railway can improve the quality of the urban environment through the mediating effect of improving the efficiency of urban factor allocation, that is, the opening of high-speed railway has the dual positive effects of economic efficiency and environmental quality improvement.*

## 4. Empirical Strategies

### 4.1. Construction of a Quantitative Evaluation Model and Variable Selection of Factor Allocation Efficiency between Prefecture-Level Cities in China

#### 4.1.1. Variable Description

In this paper, it is first necessary to construct a theoretical model to quantitatively evaluate the level of factor misallocation between prefecture-level cities in China and construct factor allocation efficiency variables for checking the three theoretical hypotheses proposed in this paper empirically. The variables used for the quantitative evaluation of the efficiency of factor allocation between prefecture-level cities in China mainly include the annual GDP, labor input, and capital stock of prefecture-level cities. In this paper, the output variable GDP of prefecture-level cities is deflated by the GDP deflator index in the base period of 2006, and it is used to represent the actual output of prefecture-level cities ($Y_{it}$). The capital stock variable ($K_{it}$) of prefecture-level cities is calculated by the perpetual inventory method, and the specific calculation formula is: $K_{t}=I_{t}/P_{t}+\left(1-\delta_{t}\right)K_{t-1}$, which $I_{t}$ indicates the investment amount of fixed assets, $P_{t}$ is the fixed asset investment price index, and $\delta_{t}$ is the current depreciation rate, which is set to 9.6% with reference to the practice of Bai and Liu [36]. $K_{t-1}$ is the stock of fixed capital in the previous period, the stock of fixed capital in the base period of 2006 is calculated using the following formula: $K_{2006}=\left(\frac{I_{2006}}{\bar{g}+\bar{\delta }}-I_{2006}\right)/\left(1-\delta_{2006}\right)$, where $\bar{g}$ and $\bar{\delta }$ respectively represent the average GDP growth rate and average depreciation rate of each prefecture-level city from 2006 to 2019. The above data is mainly from the *China City Statistical Yearbook*. The labor input ($L_{it}$) variable is expressed by the number of laborers and employment in prefecture-level cities over the years.

#### 4.1.2. Construction of a Quantitative Evaluation Model of Factor Allocation Efficiency between Prefecture-Level Cities in China

This paper refers to the model construction idea of Jin [37], assuming that the economic sector is composed of prefecture-level city departments and that the output of prefecture-level city departments cooperates with each other to obtain the total output *Y*, that is, *Y* is the CES production function of $Y_{i}$ (the annual output of the prefecture-level city i), specifically expressed as $Y=\sum_{i=1}^{N}{\left(\theta_{i}Y_{i}^{\sigma }\right)}^{1/\sigma }$, where $\sum_{i=1}^{N}\theta_{i}=1$, *θ_i_* represents the weight of the output of the prefecture-level city *i* in the total output production process. Continuing to assume that the production function of the prefecture-level city sector is $Y_{i}=A_{i}K_{i}^{\alpha }L_{i}^{1-\alpha }$, $K=\sum_{i=1}^{N}K_{i}$, $L=\sum_{i=1}^{N}L_{i}$, and that the proportion of capital input factor and labor input factor of each prefecture-level city sector is $k_{i}=\frac{K_{i}}{K},\;l_{i}=\frac{L_{i}}{L}$.

This gives us the formula for calculating the overall total factor productivity of the economy:

(1) $$TFP={\left(\sum_{i=1}^{N}\theta_{i}Y_{i}^{\sigma }\right)}^{1/\sigma }/\left(K^{\alpha }L^{1-\alpha }\right)={\left[\sum_{i=1}^{N}\theta_{i}{\left(A_{i}k_{i}^{\alpha }l_{i}^{1-\alpha }\right)}^{\sigma }\right]}^{1/\sigma }$$

Further, assume that the factor prices of a prefecture-level city i are $\tau_{i}^{k}r$, $\tau_{i}^{l}w$, which represent the price distortion coefficients of capital and labor factors, respectively. Then the profit maximization problem of total output is: $\underset{Y_{i}}{max}\left\{P{\left(\sum_{i=1}^{N}\theta_{i}Y_{i}^{\sigma }\right)}^{1/\sigma }-\sum_{i=1}^{N}P_{i}Y_{i}\right\}$, and the profit maximization problem of the output of each prefecture-level city sector is: $\underset{K_{i},L_{i}}{max}\left\{P_{i}A_{i}K_{i}^{\alpha }L_{i}^{1-\alpha }-\tau_{i}^{k}rK_{i}-\tau_{i}^{l}wL_{i}\right\}$. We can sort out the proportion of capital input proportion ($k_{i}$) and labor input proportion ($l_{i}$) of the prefecture-level city sector in a distorted state, $k_{i}=\frac{K_{i}}{K},\;l_{i}=\frac{L_{i}}{L}$:

(2) $$k_{i}=\frac{\theta_{i}^{\frac{1}{1-\sigma }}{\bar{A}}_{i}{}^{\frac{\sigma }{1-\sigma }}\tau_{i}^{k-1}}{\sum_{i=1}^{N}\theta_{i}^{\frac{1}{1-\sigma }}{\bar{A}}_{i}{}^{\frac{\sigma }{1-\sigma }}\tau_{i}^{k-1}},l_{i}=\frac{\theta_{i}^{\frac{1}{1-\sigma }}{\bar{A}}_{i}{}^{\frac{\sigma }{1-\sigma }}\tau_{i}^{l-1}}{\sum_{i=1}^{N}\theta_{i}^{\frac{1}{1-\sigma }}{\bar{A}}_{i}{}^{\frac{\sigma }{1-\sigma }}\tau_{i}^{l-1}}$$

Thereinto, $\bar{A_{i}}=A_{i}{\left(\tau_{i}^{k}\right)}^{-\alpha }{\left(\tau_{i}^{l}\right)}^{\alpha -1}$. Substituting the expression (2) of the capital and labor factor input proportion of each prefecture-level city into Equation (1), the overall total factor productivity of the economy in a distorted state can be obtained:

(3) $$TFP={\left(\sum_{i=1}^{n}\theta_{i}^{\frac{1}{1-\sigma }}{\bar{A_{i}}}^{\frac{\sigma }{1-\sigma }}\right)}^{\frac{1-\sigma }{\sigma }}\times {\left(\frac{\sum_{i=1}^{n}\theta_{i}^{\frac{1}{1-\sigma }}{\bar{A_{i}}}^{\frac{\sigma }{1-\sigma }}\tau_{i}^{K-1}}{\sum_{i=1}^{n}\theta_{i}^{\frac{1}{1-\sigma }}{\bar{A_{i}}}^{\frac{\sigma }{1-\sigma }}}\right)}^{-\alpha }\times {\left(\frac{\sum_{i=1}^{n}\theta_{i}^{\frac{1}{1-\sigma }}{\bar{A_{i}}}^{\frac{\sigma }{1-\sigma }}\tau_{i}^{L-1}}{\sum_{i=1}^{n}\theta_{i}^{\frac{1}{1-\sigma }}{\bar{A_{i}}}^{\frac{\sigma }{1-\sigma }}}\right)}^{\alpha -1}$$

Therefore, it can be obtained that when there is no misallocation of resources between the prefecture-level city departments, that is, in the effective state ($\tau_{i}^{k}=\tau_{i}^{l}=1$), the proportion of effective capital investment of each prefecture-level city department ($k_{i}^{*}$) and the proportion of effective labor input ($l_{i}^{*})$:

(4) $$k_{i}^{*}=\frac{\theta_{i}^{\frac{1}{1-\sigma }}A_{i}{}^{\frac{\sigma }{1-\sigma }}}{\sum_{i=1}^{N}\theta_{i}^{\frac{1}{1-\sigma }}A_{i}{}^{\frac{\sigma }{1-\sigma }}},l_{i}^{*}=\frac{\theta_{i}^{\frac{1}{1-\sigma }}A_{i}{}^{\frac{\sigma }{1-\sigma }}}{\sum_{i=1}^{N}\theta_{i}^{\frac{1}{1-\sigma }}A_{i}{}^{\frac{\sigma }{1-\sigma }}}$$

Substituting Equation (4) into Equation (1), the overall total factor productivity of the economy in the effective state is obtained:

(5) $$TFP^{*}={\left(\sum_{i=1}^{N}\theta_{i}^{\frac{1}{1-\sigma }}A_{i}^{\frac{\sigma }{1-\sigma }}\right)}^{\frac{1-\sigma }{\sigma }}$$

As mentioned earlier, this paper attributes the resource misallocation caused by differences between prefecture-level cities to total factor productivity losses. The degree of total factor productivity loss caused by the misallocation of total factors between departments in prefecture-level cities is:

(6) $$d=\frac{TFP^{*}}{TFP}-1$$

This paper continues to make the labor price distortion coefficient $\tau_{i}^{l}$ and the capital price distortion coefficient $\tau_{i}^{k}$ equal to 1, and then examines the degree of total factor productivity loss caused by capital misallocation and labor misallocation, respectively. The first-order condition for profit maximization in prefecture-level city departments can be obtained as follows: $\tau_{i}^{K}\propto \frac{Y_{i}^{nor}}{K_{i}}$, $\tau_{i}^{L}\propto \frac{Y_{i}^{nor}}{L_{i}}$, calculation formula of $\theta_{i}$ obtained according to the first-order condition of profit maximization of overall output is: $\theta_{i}=\frac{1}{T}\sum_{t=1}^{T}\frac{P_{i}\left(t\right){\left[Y_{i}^{nor}\left(t\right)/P_{i}\left(t\right)\right]}^{\sigma }}{\sum_{i=1}^{N}P_{i}\left(t\right){\left[Y_{i}^{nor}\left(t\right)/P_{i}\left(t\right)\right]}^{\sigma }}$. This article continues with Brandt and Zhu (2010) for capital-output elasticity =0.45 and with Brandt et al. [38] for =1/3.

### 4.2. Empirical Model Construction and Variable Description of the Relationship between High-Speed Railway Opening, Inter-Regional Factor Allocation Efficiency, and Urban Environmental Pollution

#### 4.2.1. Variable Description

This paper uses the panel data of prefecture-level cities in China from 2006 to 2019 to construct an econometric model, empirically analyzes the internal impact mechanisms of high-speed railway opening, inter-regional factor allocation efficiency, and urban environmental pollution, and tests the three hypotheses proposed above. All data are from the *China City Statistical Yearbook* unless otherwise noted.

High-speed railway opening is variable. This paper mainly selects the virtual variables of high-speed railway opening (HSR) and the number of high-speed railway lines (*HSRsum)* to measure the opening of high-speed railway in a city. If city *i* has a high-speed railway line opened in year *t*, set the high-speed railway opening dummy variable (*HSR)* to 1, otherwise it is zero. The number of high-speed railway lines (*HSRsum*) indicates the number of high-speed railway lines that have been opened in city *i* in year *t*. The data for this variable are mainly obtained from the official documents of the State Railway Administration, the *China Railway Yearbook*, the website of the China State Railway Croup Co., Ltd. (Beijing, China), as well as the information on the 12306 website and www.qunar.com (accessed on 19 November 2021).

Inter-regional factor allocation efficiency is variable. In this paper, while using the above model to calculate the overall factor misallocation and capital and labor misallocation between prefecture-level cities in China, it continues to construct the factor misallocation index variables at the level of the prefecture-level city. Drawing on the above ideas, the total misallocation of factors, capital misallocation, and labor misallocation of prefecture-level city *I* are respectively: $mis_{i}=\left|\frac{Y^{*}}{Y}-1\right|$, $misk_{i}=\left|\frac{Y^{k*}}{Y}-1\right|$, $misl_{i}=\left|\frac{Y^{l*}}{Y}-1\right|$. Among them, $Y^{*},Y^{k*},\;Y^{l*}$, respectively represent the output level of prefecture-level cities when the prefecture-level city *i* is invested according to the proportion of all effective factors, only effective capital input, and only effective labor input, *Y* represents the actual output level of prefecture-level cities, and the above expression is the level of deviation between the output of prefecture-level cities in a valid state and the actual output of prefecture-level cities, which is used as a proxy indicator of the factor allocation efficiency of prefecture-level cities. The higher value indicates a higher level of factor misallocation and lower allocation efficiency.

Urban environmental pollution variables. Drawing on the research ideas of Shi et al. [39], this paper selects urban wastewater discharge (*ww*) and exhaust emission (*so*) to measure the level of urban environmental pollution. 

The foreign direct investment variable (*fdi*) indicates the proportion of FDI in urban GDP. The industrial structure variable (*str*) is expressed by the proportion of the output value of the tertiary industry to the output value of the secondary industry, and the higher the value of the variable, the higher the level of industrial structure upgrading. The technological innovation variable (*cx*) is expressed by the number of patents per capita. The urban income level variable (*rgdp*) is expressed by urban GDP per capita. Population density (*pop*) is expressed by dividing the number of urban populations by the area of the city’s administrative divisions. The level of human capital (*hc*) is expressed by the number of college students per 10,000 people.

Referring to the control variable selection method of previous research literature, the control variables selected in this paper mainly include: the urbanization rate (*urb*), expressed as the proportion of non-agricultural population to total population; the level of financial development (*fin*), expressed as the proportion of the balance of urban deposits and loans to the city’s GDP; the level of road infrastructure construction (*inf*), expressed as the number of highway miles per square kilometer of the city; and the opening degree (open), expressed as the proportion of total imports and exports to the GDP of this city.

#### 4.2.2. Construction of an Empirical Model on the Relationship between High-Speed Railway Opening, Factor Allocation Efficiency, and Urban Environmental Pollution

This paper uses the causal steps approach to empirically test the mediating effect designed in the hypothesis. The baseline model and mediation effect model for testing Hypothesis 1 are set as follows:

(7) $$mis_{it}\&misk_{it}\&misl_{it}=\alpha +\beta HSR_{it}\&HSRsum_{it}+\gamma X_{it}+u_{i}+v_{t}+\varepsilon_{it}$$

(8) $$\;mis_{it}\&misk_{it}\&misl_{it}=\alpha +\beta HSR_{it}\&HSRsum_{it}+\delta cx_{it}\left(fdi_{it}\;\&\;pop_{it}\right)+\gamma X_{it}+u_{i}+v_{t}+\varepsilon_{it}$$

(9) $$cx_{it}\left(fdi_{it}\;\&\;pop_{it}\right)=\alpha +\beta HSR_{it}\&HSRsum_{it}+\gamma X_{it}+u_{i}+v_{t}+\varepsilon_{it}$$

The explained variable is the factor misallocation variable ($mis_{it}\&misk_{it}\&misl_{it}$), the core explanatory variable is the high-speed railway opening variable ($HSR_{it}\&HSRsum_{it}$), and the intermediary variables are the technological innovation variable (*cx_it_*), the foreign direct investment variable (*fdi_it_*), and the population agglomeration variable (*pop_it_*). $X_{it}$ is a series of control variables. $v_{t}$ is a fixed effect in time, $u_{i}$ is an entity’s fixed effect, and $\varepsilon_{it}$ is an error term. 

In the model (7), the expected sign of the variable coefficient of high-speed railway opening is negative, indicating that the opening of high-speed railways can significantly reduce the level of inter-regional factor misallocation in cities. The expected signs of the coefficients of the above mediating variables in model (8) are all negative, indicating that the opening of high-speed railways reduces the level of urban factor misallocation by promoting technological innovation, foreign investment attraction, and population agglomeration. If the impact of high-speed railway opening on the efficiency of urban factor allocation is mainly generated through the above intermediary mechanism, it should be satisfied: First, the high-speed railway opening variable has a negative and significant effect on the urban factor misallocation variable in Equation (7). Secondly, the high-speed railway opening variable has a positive and significant effect on the intermediary variable in Equation (9). Finally, in Equation (8), the intermediary variable has a negative and significant effect on the urban factor misallocation variable, and after the introduction of the intermediary variable, the coefficient of the information benefiting urban policy variable changes from negative and significant in Equation (7) to insignificant in Equation (8), or the coefficient of the high-speed railway opening variable in Equation (8) is still negative and significant, but the absolute value of the coefficient decreases.

Continuing the above empirical test ideas, this paper continues to set the baseline model and mediation effect model for testing Hypothesis 2 as follows:

(10) $$ww_{it}\&so_{it}=\alpha +\beta mis_{it}+\gamma X_{it}+u_{i}+v_{t}+\varepsilon_{it}$$

(11) $$\;ww_{it}\&so_{it}=\alpha +\beta mis_{it}+\delta str_{it}\left(agdp_{it}\;\&\;hc_{it}\right)+\gamma X_{it}+u_{i}+v_{t}+\varepsilon_{it}$$

(12) $$str_{it}\left(agdp_{it}\;\&\;hc_{it}\right)=\alpha +\beta mis_{it}+\gamma X_{it}+u_{i}+v_{t}+\varepsilon_{it}$$

The explained variable is the urban environmental pollution variable ($ww_{it}\&so_{it}$), the core explanatory variable is the urban factor misallocation variable ($mis_{it}$) and the mediating variables are the industrial structure upgrading variable (*str_it_*), the per capita income level variable (*agdp_it_*), and the human capital agglomeration variable (*hc_it_*). $X_{it}$ is a series of control variables that is consistent with the above. In the model (10), the sign of the coefficient of the urban factor misallocation variable is expected to be positive, indicating that the improvement of urban factor allocation efficiency can significantly reduce the city’s environmental pollution levels. The expected signs of the coefficients of the above intermediary variables in the model (11) are all negative, indicating that the improvement of urban factor allocation efficiency can reduce urban environmental pollution by promoting industrial structure upgrades, per capita income level improvements, and human capital agglomeration. If the impact of urban factor allocation efficiency on urban environmental pollution is mainly generated through the above intermediary mechanism, it should be satisfied: First, the urban factor allocation efficiency variable (namely, the element misallocation variable) has a positive and significant impact on urban environmental pollution variables in Equation (10); secondly, the urban factor misallocation variable has a negative and significant effect on the mediation variable in Equation (12); Finally, in Equation (11), the mediating variable has a negative and significant effect on the urban environmental pollution variable, and after introducing the intermediary variables, the coefficient of the urban element misallocation variable changes from positive and significant in Equation (10) to insignificant in Equation (11), or the coefficient of the urban element misallocation variable in Equation (11) is still positive and significant, but the absolute value of the coefficient decreases. 

Finally, the baseline model and mediation effect model for testing Hypothesis 3 are set up along the same lines:

(13) $$ww_{it}\&so_{it}=\alpha +\beta HSR_{it}\&HSRsum_{it}+\gamma X_{it}+u_{i}+v_{t}+\varepsilon_{it}$$

(14) $$\;ww_{it}\&so_{it}=\alpha +\beta HSR_{it}\&HSRsum_{it}+\delta mis_{it}+\gamma X_{it}+u_{i}+v_{t}+\varepsilon_{it}$$

(15) $$mis_{it}=\alpha +\beta HSR_{it}\&HSRsum_{it}+\gamma X_{it}+u_{i}+v_{t}+\varepsilon_{it}$$

The meaning of the coefficient characteristics of the above model is similar to the above and will not be repeated here.

#### 4.2.3. The PSM-DID Method Is Used to Eliminate the Selective Bias Problem of High-Speed Railway Openings

When selecting the target city, it is likely that the high-speed railway will not be randomly selected, which may lead to the selectivity bias of the sample. In order to effectively solve the problem of possible selection bias in the opening of high-speed railways and effectively avoid the systematic differences in the factor misallocation variables and the change trend of environmental pollution variables between high-speed railway opening cities and non-high-speed railway opening cities, this paper continues to use the propensity score matching method (*PSM-DID*) to reselect the control group samples so that the control group and the treatment group samples can be directly compared and analyzed. In this paper, the *Probit* model is mainly used to estimate the propensity scores of the treatment group variables and the control group variables, and finally, we find the samples of non-high-speed railway opening cities that are similar to the high-speed railway opening cities. The *Probit* model used in the article is as follows:

(16) $$HSR_{i,t}=\alpha_{0}+\gamma X_{i,t}+Year+\varepsilon_{i,t}$$

$HSR_{i,t}$ indicates the dummy variable of high-speed railway opening, $HSR_{i,t}=1$ if it is a high-speed railway opening city, and $HSR_{i,t}=0$ if it is a non-high-speed railway opening city. $X_{i,t}$ is a series of control variables, this paper mainly selects the degree of urban openness (*open*), infrastructure construction level (*inf*), financial development variable (*fin*), and urbanization level (*urb*) in the *Probit* regression and propensity matching scores. 

**Figure 2:**
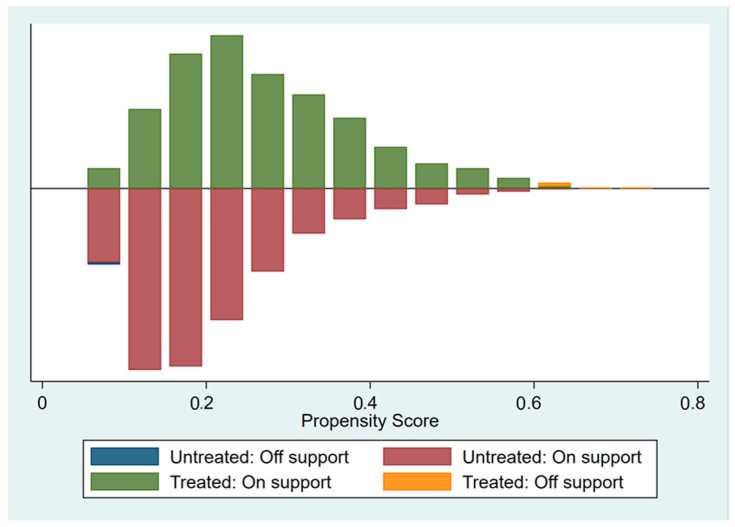
Common value range of propensity score.

**Figure 3:**
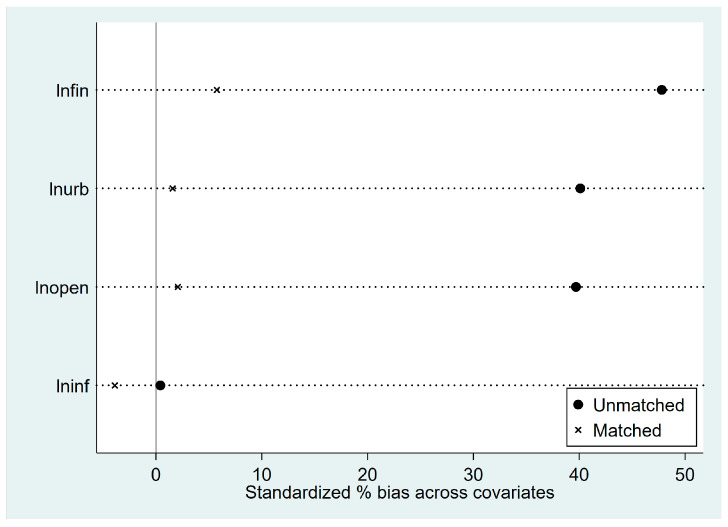
Standardized deviation of each variable.

**Figure 4:**
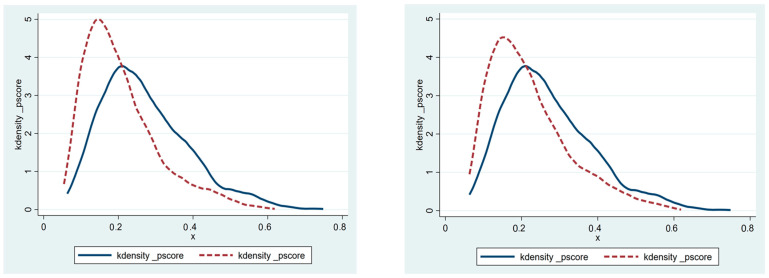
Density map before and after propensity score matching.

Before the propensity score matching can be performed, the sample data needs to meet the “overlap assumption”, that is, that the propensity score of the control group and the treatment group samples have a common value. It can be seen from Figure 2 that the common propensity scores of the treatment group and control group are significant, and most of the treatment and control group sample propensity scores are within the common value range. After the propensity score matching, it can be seen from Figure 3 that the standardization bias of most variables has been significantly reduced, which shows that the propensity score matching method used in this paper can effectively eliminate the sample selection bias problem and clear the obstacle for the next step of PSM-DID regression analysis. The density curves of the two groups of city samples before and after propensity score matching can be seen in Figure 4, and before the propensity score matching, the propensity score density curves of the treatment group and control group samples have a large deviation, and after the propensity score matching, the propensity score probability density curves of the two groups of cities have been very similar, indicating that the propensity score matching eliminates most of the choice bias.

### 4.3. Descriptive Statistical Results for Primary Variables

Table 1 provides sample information on the mean, standard deviation, maximum, and minimum values of the main variables from 2006 to 2019 at the prefecture-level city level. Among them, the average misallocation of factors (capital factors and labor factors) in cities where high-speed rail is open is lower than that in cities that have not opened high-speed rail. On the whole, the average wastewater discharge and exhaust emission in cities without high-speed railways are higher than those in cities with high-speed railways. It can be preliminarily inferred that the opening of high-speed railways does cause a significant difference in the efficiency of factor allocation and the level of urban environmental pollution in the city. From the six intermediary variables selected in this paper, the per capita GDP, foreign direct investment, human capital level, population density, industrial structure upgrading level, and technological innovation output of cities with high-speed railway opening are also significantly higher than those of cities without high-speed railway.

## 5. Empirical Results and Analysis

### 5.1. Quantitative Assessment Results and Characteristic Analysis of Factor Misallocation between Prefecture-Level Cities in China

According to the mathematical model constructed in this paper, the misallocation results between prefecture-level cities are shown in Table 2 by using the data of prefecture-level cities in China from 2006 to 2019. It can be seen from Table 2 that the average annual misallocation level of factor misallocation between prefecture-level cities in China was as high as 52.5% between 2006 and 2019. That is, the factor misallocation between China’s prefecture-level cities caused the loss of China’s total economic output or total factor productivity to be 52.5% during the sample period. Among them, the average misallocation of labor factors was 23.16%. The average misallocation of capital factors was 18.69%, and on the whole, the misallocation of factors between prefecture-level cities in China was mainly caused by labor misallocation.

**Figure 5:**
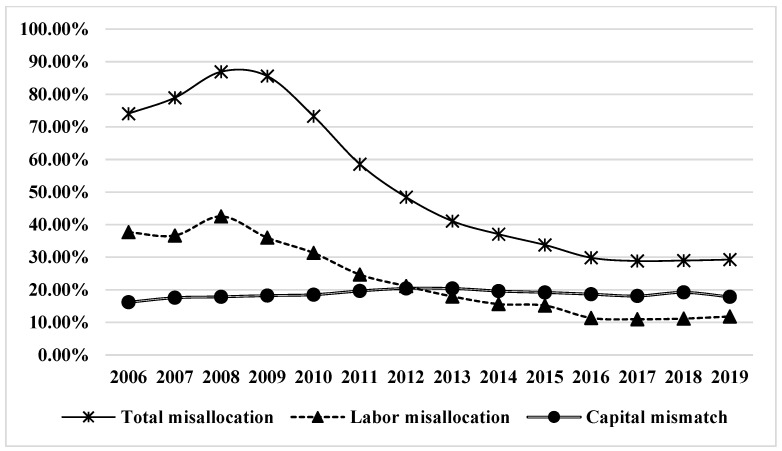
A schematic diagram of the misallocation level of factors between prefecture-level cities in China from 2006 to 2019. Note: The authors compiled based on the data in Table 2.

In order to analyze the trend of factor misallocation between prefecture-level cities in China more clearly, this paper continues to plot the data in Table 2 as Figure 5. It can be seen from Figure 5 that from 2006 to 2008, the misallocation of factors between prefecture-level cities in China was at a high level and continued to rise, and since 2008, the misallocation level of factors between prefecture-level cities has been decreasing year by year. It happened that between 2008 and 2009, China’s high-speed railway mileage began to show a rapid development trend, and the annual new mileage of China’s high-speed railway began to increase from 631 km in 2008 to 2349 km in 2009, and the total mileage of China’s high-speed railway has exceeded 35,000 km, ranking first in the world. The rapid decline in the misallocation level of factors between prefecture-level cities in China is highly synchronized with the rapid rise in the mileage of China’s high-speed railway lines. Therefore, it is necessary to deeply explore the intrinsic relationship and influence mechanism between the opening of high-speed railways and the efficiency of factor allocation in prefecture-level cities. From the perspective of subdivision factor misallocation, between 2006 and 2012, labor misallocation was significantly higher than capital misallocation, and China’s prefecture-level inter-city factor misallocation was mainly caused by labor misallocation. Moreover, the trend of labor misallocation is highly consistent with the trend of total misallocation, and both increase and then decrease with 2008 as the inflection point. With the continuous decline of the level of labor factor misallocation, after 2012, capital factor misallocation gradually exceeded labor factor misallocation, becoming an important source of factor misallocation between prefecture-level cities in China.

### 5.2. Empirical Results of the Internal Impact Mechanism of High-Speed Railway Opening, Urban Factor Allocation Efficiency, and Urban Environmental Pollution

#### 5.2.1. Empirical Test Results and Analysis of the Intermediary Mechanism of High-Speed Railway Opening Affecting the Efficiency of Urban Factor Allocation

In this paper, a two-way fixed-effects model was used for baseline regression estimation. The results of the test for Hypothesis 1 are reported in Table 3 and Table 4. Among them, Table 3 mainly reports the test results of the impact of high-speed railway opening on the efficiency of factor allocation in prefecture-level cities. It can be seen from Table 3 that the regression coefficients of total misallocation, capital misallocation, and labor misallocation of prefecture-level cities are significantly negative at the 1% significance level, whether the high-speed railway opening variable or the high-speed railway total line number variable is used, which indicates that the high-speed railway opening can significantly reduce the factor misallocation of China’s prefecture-level cities and has a significant negative impact on capital misallocation and labor misallocation. From the perspective of influence, the corrective effect of the opening of high-speed rail on capital misallocation is stronger than that of labor misallocation.

Next, this paper will empirically test the mediating effect mechanism of high-speed railway openings on the factor allocation efficiency of prefecture-level cities according to models (7)–(9). Firstly, let’s look at the mechanism of the mediation effect of the opening of high-speed railways on the total factor misallocation in prefecture-level cities. The regression results show that in columns 2 and 3 of the upper part of Table 4, the coefficients of the high-speed railway opening variable are negative and significant at the level of 1%, indicating that the opening of high-speed railways can indeed reduce the total factor misallocation in prefecture-level cities. In addition, according to the analysis of Hypothesis 1, it is also necessary to examine whether the opening of a high-speed railway mainly corrects the total factor misallocation of prefecture-level cities by promoting the improvement of urban scientific and technological innovation capabilities, attracting foreign direct investment, and improving the level of population agglomeration. It can be seen from columns 1, 4, and 6 in the upper part of Table 4 that the coefficients of the high-speed railway opening variables are all positive and significant at 1%, indicating that the opening of a high-speed railway can indeed improve the city’s scientific and technological innovation capabilities, accelerate the attraction of foreign direct investment, and increase the degree of population agglomeration. Columns 3, 5, and 7 of Table 4 introduce high-speed railway opening variables, urban science and technology innovation variables, foreign direct investment variables, and population agglomeration variables on the total factor misallocation in prefecture-level cities. The regression results showed that the coefficients of the technological innovation variable, foreign direct investment variable, and population agglomeration variable were all negative and significant at the 1% level, while the coefficient of the high-speed railway opening variable was still negative and significant at the 1% level, but the absolute value of the coefficient was lower than the one in the second column, indicating that the improvement of technological innovation ability, attracting foreign direct investment, and improving population agglomeration as the intermediary mechanism of high-speed railway opening affecting the efficiency of urban total factor allocation have been supported by empirical results.

The middle and lower parts of Table 4 continue to empirically test the effectiveness of the intermediary mechanism that affects the efficiency of capital factor allocation and labor factor allocation in prefecture-level cities. After replacing the factor misallocation variables from the total factor misallocation in prefecture-level cities with capital misallocation and labor misallocation, the effectiveness of improving urban technological innovation capabilities, attracting foreign direct investment, and improving population agglomeration as intermediary mechanisms of high-speed railway opening affecting the urban capital factor allocation efficiency and labor factor allocation efficiency is re-examined. It can be seen from the empirical regression results in Table 4 that the empirical test results are basically consistent with the factor misallocation variable when the factor misallocation variable is the total factor misallocation variable of the prefecture-level city, and this will not be elaborated here. That is, the empirical test results show that the opening of high-speed railways can improve the efficiency of capital and labor factor allocation in prefecture-level cities by improving the city’s technological innovation ability, attracting foreign direct investment, and increasing population agglomeration, and the intermediary mechanism has a significant impact on high-speed railway opening and total factor misallocation, capital factor misallocation, and labor factor misallocation in prefecture-level cities.

Therefore, the content of Hypothesis 1 is fully supported by the empirical results, i.e., the opening of high-speed railways can indeed promote the improvement of urban factor allocation efficiency through the technological innovation effect, the foreign investment attraction effect, and the population agglomeration effect.

#### 5.2.2. Empirical Test Results and Analysis of the Impact Mechanism of the Urban Factor Allocation Efficiency Improvement for Urban Environmental Pollution

Next, this paper continues to empirically test the content of Hypothesis 2 according to the ideas of models (10)–(12), that is, the influence mechanism of urban factor allocation efficiency improvement on urban environmental pollution. Columns 2 and 4 of Table 5 are the regression results for the model (10). When the explained variables were exhaust emission (*so*) and wastewater discharge (*ww*), the coefficients of urban factor misallocation variables were positive and significant at levels of 5% and 1%, respectively. It shows that improving the efficiency of urban factor allocation can indeed reduce urban environmental pollution. In addition, according to the analysis of Hypothesis 2, it is also necessary to investigate whether the improvement of urban factor allocation efficiency is mainly achieved through promoting the upgrading of urban industrial structure, the improvement of urban per capita income level, and the improvement of the human capital agglomeration level to reduce urban environmental pollution. It can be seen from the first column in the upper part of Table 5 that the coefficient of the urban factor misallocation variable is negative and significant at the level of 1%, indicating that the improvement of urban factor allocation efficiency can significantly promote the upgrading of urban industrial structure. Columns 3 and 5 in the upper part of Table 5 are the regression results of the model (11). The urban factor misallocation variable and the industrial structure upgrading variable were introduced into the model at the same time to investigate the effectiveness of the mediating effect of industrial structure upgrading. The empirical test results show that the variable coefficients of industrial structure upgrading are negative and significant at the level of 1%, i.e., urban industrial structure upgrading can significantly reduce urban environmental pollution. After the introduction of the industrial structure upgrading variable, the urban factor misallocation variable is still positive and at least 5% significant, but the absolute value of the coefficient is smaller than that without the industrial structure upgrading variable. It shows that industrial structure upgrading as an intermediary mechanism for urban factor allocation efficiency to affect urban environmental pollution is supported by empirical results. This paper continues to empirically examine the other two mediating mechanisms that affect urban environmental pollution due to the improvement of urban factor allocation efficiency: the revenue-boosting effect and the human capital agglomeration effect. The empirical results in the middle and lower parts of Table 5 show that the two mediating mechanisms are also supported by the empirical results. 

Therefore, the content of Hypothesis 2 is fully supported by the empirical results, i.e., the improvement of urban factor allocation efficiency can promote the improvement of urban environmental quality through the industrial structure optimization effect, revenue-boosting effect, and human capital agglomeration effect.

#### 5.2.3. The Impact of High-Speed Railway Opening on Urban Environmental Pollution: An Empirical Test Based on the Impact Mechanism of Urban Factor Allocation Efficiency

If Hypotheses 1 and 2 are true at the same time, then Hypothesis 3 must also be true. This paper continues to use the model (13)–(15) to empirically test whether the content of Hypothesis 3 is valid, that is, whether the opening of high-speed railways can achieve the effect of improving urban environmental pollution by improving the efficiency of urban factor allocation. As can be seen from the first column of Table 6, the variable of high-speed railway opening is negative and significant at the level of 1%, indicating that the opening of high-speed railways can significantly reduce the urban factor misallocation and improve the efficiency of urban factor allocation. The regression results in columns 2 and 4 of Table 6 show that the opening of high-speed railways can significantly reduce urban wastewater discharge and exhaust emissions and improve urban environmental quality. Columns 3 and 5 of Table 6 introduce the high-speed railway opening and the intermediary variable, the urban factor misallocation variable, into the model at the same time, and the coefficient of the urban factor misallocation variable is positive with at least 10% significance. After the introduction of the urban factor misallocation variable, although the high-speed railway opening variable is still significantly negative, the absolute value of the coefficient is lower than that of columns 2 and 4 in Table 6. This shows that the opening of high-speed railways can indeed reduce urban environmental pollution by improving the efficiency of urban factor allocation. Therefore, the content of Hypothesis 3 is fully supported by empirical results.

## 6. Heterogeneity Analysis

### 6.1. Analysis of City-Scale Heterogeneity

The previous analysis shows that the opening of high-speed railways can significantly reduce urban factor misallocation and urban environmental pollution. For cities of different scales, do the resource allocation effect and the environmental governance effect of high-speed railway openings exist? If so, what is the difference between the resource allocation effect and the environmental governance effect of high-speed railway openings in cities of different scales? From the perspective of city scale, larger cities are more likely to form economic agglomeration effects and can then have higher resource allocation efficiency and a higher energy level industrial structure, which is also conducive to reducing urban environmental pollution. However, at the same time, large-scale cities can also produce congestion effects, resulting in “big city disease” and other problems, which will aggravate the urban factor misallocation and increase the level of environmental pollution. Therefore, the resource allocation effect and environmental governance effect of high-speed rail opening on cities of different scales depend on the rebalancing of the above two effects. Theoretically, the opening of high-speed railways can enhance the economic agglomeration effect of cities and also reduce the congestion effect caused by excessive urban scale by improving the accessibility of cities and the convenience of population movement. From this point of view, the above-mentioned double positive effect of the opening of high-speed railways is likely to be more significant for large-scale cities. Because the economic agglomeration effect and urban congestion effect of small-scale cities are relatively weak, the marginal impact of high-speed railway opening on them may be relatively small, so the factor allocation effect and environmental governance effect of high-speed railway opening may be relatively weaker than those of large cities. This paper defines cities with a population greater than or equal to 5 million as large cities, cities with a population of 3 million to 5 million as medium-sized cities; and cities with a population of less than 3 million as small cities. Table 7 shows the impact of the opening of high-speed railways on the factor allocation efficiency of cities of different scales. Table 7 indicates that the factor allocation effect of high-speed railway opening increases with the expansion of city scale, the coefficient of high-speed railway opening variable in large city samples is negative and significant at the level of 1%, the coefficient of high-speed railway opening variable in the sample of medium-sized cities is negative and significant at the level of 5%, but the absolute value of the coefficient is significantly lower than that of large cities, and the coefficient of high-speed railway opening variable in the small city sample was negative but not significant. This also confirms the view that the larger the scale of the city, the stronger the factor allocation efficiency optimization effect of a high-speed railway opening.

Considering that the impact of factor flow and industrial agglomeration caused by the opening of high-speed rail on the environment may vary significantly with the size of cities, we further empirically investigated the impact of the opening of high-speed rail on the environment of cities of different sizes [40]. In terms of the environmental governance effect of the opening of high-speed railways, the empirical test results of the impact of high-speed railway opening on environmental pollution in cities of different scales are shown in Table 8. It can be seen from Table 8 that the absolute value of the high-speed railway opening coefficient is the largest and most significant in the sample of medium-sized cities, whether it is wastewater discharge or exhaust emission. The coefficients of the high-speed railway opening variable were significant in the large city sample, and the absolute value of the coefficient was relatively small compared with that of medium-sized cities. The opening of high-speed railways has a significant and negative impact on the exhaust emissions of small cities, but the absolute value of the coefficient is the smallest compared with that of large and medium-sized cities, and the significance level is also low. In addition, the opening of high-speed railways does not have a significant impact on wastewater discharge in small cities.

### 6.2. Analysis of Heterogeneity of Urban Characteristics

From the perspective of heterogeneity of urban characteristics, it is beneficial to examine which urban development characteristics can strengthen the urban environmental governance effect of high-speed railway openings, which is beneficial to the planning and construction of future high-speed railway networks and the high-quality development of cities along the route. The focus on introducing and studying which urban development characteristics have practical significance and policy value needs to be considered. To this end, this paper makes the following considerations: In addition to the need for multiple economic entities within the city to independently reduce pollutant emissions when carrying out economic activities, urban environmental governance also requires the government to carry out public environmental pollution control by investing human, financial, and material resources. In order to enhance the policy value of introducing urban characteristics, this paper focuses on the four characteristics of urban financial pressure, financial development, human capital, and urban administrative level. In the specific empirical process, this paper divides the fiscal pressure variable and the human capital level variable into high fiscal pressure cities and low fiscal pressure cities, high human capital cities and low human capital cities, and then divides the sample into provincial capital cities and non-provincial capital cities according to whether the city is a provincial capital, so as to investigate the impact of urban administrative level heterogeneity. The empirical test results show in Table 9 that the opening of high-speed railways has a significant effect on the waste water discharge of cities with high fiscal pressure, but the absolute value of the coefficient is significantly lower than that of cities with low fiscal pressure, and the impact of high-speed railway openings on the exhaust emissions of cities with high fiscal pressure is not significant, while the impact on the exhaust emissions of cities with low fiscal pressure is negative and significant at a level of 1%. This shows that the stronger the financial strength of the city, the more the government can carry out urban environmental governance with the help of the favorable conditions created by the opening of high-speed railway, which will also make the environmental governance effect of the opening of high-speed railway stronger and smoother the intermediate impact mechanism. The environmental governance effect of high-speed rail in cities with high human capital levels is very significant, while the environmental governance effect of high-speed rail in cities with low human capital levels is not significant. On the one hand, higher human capital agglomeration provides governance support for enterprise green technology updating and urban environmental governance. The opening of high-speed railways can further strengthen and amplify the intellectual resource advantages of cities with abundant human capital and successfully transform them into significant environmental governance results. Cities with low levels of human capital, after the opening of high-speed railways, are likely to exacerbate the local brain drain in the process of strengthening the flow of human capital, so the opening of high-speed railways may also trigger a “polarization effect” between different types of cities. Finally, the opening of high-speed railways in provincial capitals and non-provincial capitals can significantly reduce urban environmental pollution, but the environmental governance effect of high-speed railways in provincial capitals is stronger than that in non-provincial capitals. The above research shows that the more abundant the financial strength and human capital of the city and the higher the level of urban administration, the stronger the environmental governance effect of the opening of a high-speed railway.

### 6.3. Analysis of Heterogeneity in Urban Areas

China has a vast territory, and there are great differences in factor endowments, ecological environment quality, and economic development level between different regions. The difference in the level of transportation infrastructure construction, such as high-speed railways, is also obvious, and the number of high-speed railway lines opened shows the characteristics of a decreasing gradient in the eastern, central, and western regions. Therefore, the optimization effect of factor allocation efficiency and the environmental governance effect of high-speed railway opening are likely to be very different in different regional cities. In order to further identify the heterogeneous impact of high-speed railway opening on the efficiency of urban factor allocation and environmental governance in different regions, this paper further divides the urban sample into eastern, central, and western regions for empirical research, and the estimated results are shown in Table 10 and Table 11.

The estimated results in Table 10 show that there is significant regional heterogeneity in the factor allocation efficiency optimization effect of high-speed railway openings, whether it is total misallocation, capital misallocation, or labor misallocation; the factor allocation efficiency optimization effect of high-speed railway openings is the strongest and most significant in the western region, followed by the central region (the impact coefficient is significantly negative but the absolute value of the coefficient is relatively small), and the eastern region is the weakest (the impact coefficient is negative but not significant). This shows that in the future, it is urgent to further improve the relatively low level of high-speed railway infrastructure construction in the western region and promote the efficient and free flow and allocation of urban factors in the western region by further improving the high-speed railway network in the western region, so as to help the total factor productivity improvement and high-quality development of the western region’s economy.

The estimation results in Table 11 also show that there is significant regional heterogeneity in the environmental governance effect of high-speed railway opening, whether it is for urban exhaust emissions or waste water discharge. The environmental governance effect of high-speed railway opening is strongest in the western region (the coefficient is negative and significant at the level of 1%, and the absolute value of the coefficient is the largest compared with the sample coefficient in the eastern and central regions), followed by the central region (the coefficient is negative and significant at the level of 5%, but the absolute value of the coefficient is small compared with the western regions), and the eastern region is the weakest (the regression coefficient sign of the high-speed railway opening variable is uncertain and not significant).

## 7. Main Conclusions and Policy Implications

Based on the panel data of prefecture-level cities in China from 2006 to 2019, this paper uses the PSM-DID method to empirically test the intrinsic influence mechanism between the opening of high-speed railways, the efficiency of inter-regional factor allocation, and urban environmental governance. The research conclusions show that:

(1) There is serious factor misallocation among prefecture-level cities in China, and from 2006 to 2019, the factor misallocation among prefecture-level cities led to an average annual loss of total factor productivity in China’s economy of 52.5%; the average labor misallocation was 23.16%; the average capital misallocation was 18.69%; and since 2013, capital misallocation has surpassed labor misallocation as the main reason for factor misallocation among prefecture-level cities in China. In the existing literature, when measuring the level of inter-regional factor misallocation in China, most of them measured the factor misallocation among provinces in China and have not further deepened their study to include the level of factor misallocation among prefecture-level cities [37]. Compared with the level of factor misallocation among provinces in China measured by Jin [37], the level of factor misallocation among prefecture-level cities in China measured in this paper is relatively higher. Therefore, it is necessary to study the efficiency of factor allocation in China at the level of prefecture-level cities.
(2) Through the foreign investment attraction effect, the opening of a high-speed railway can promote the improvement of factor allocation efficiency among prefecture-level cities. According to previous literature, the opening of high-speed railways is conducive to attracting foreign direct investment in cities along the line, thus optimizing the local environment [30]. However, few literatures have studied the changes in factor allocation efficiency caused by changes in foreign direct investment. This conclusion enriches our understanding of the economic effects of foreign direct investment. The opening of high-speed railways can also promote the improvement of factor allocation efficiency among prefecture-level cities through the population agglomeration effect and the technological innovation effect. The above intermediary mechanisms are effective for the improvement of total misallocation, capital misallocation, and labor misallocation. Compared with the previous literature, which often studies the resource allocation effect of high-speed rail from a single intermediary mechanism [25,40], this paper analyzes the multiple intermediary mechanisms of high-speed rail affecting the efficiency of factor allocation from a more comprehensive perspective, which can help better understand the resource allocation effect of high-speed rail opening.
(3) In the previous literature, most of them have studied the factor allocation effect or environmental pollution effect of high-speed railway openings separately [6,13], and few have studied the relationship between factor allocation and environmental pollution. This paper’s conclusion shows that the improvement of factor allocation efficiency among prefecture-level cities can promote the improvement of urban environmental quality through the effects of industrial structure optimization, income improvement, and human capital agglomeration. Therefore, the opening of a high-speed railway can improve the quality of the urban environment through the mediating effect of improving the efficiency of urban factor allocation; that is, the opening of a high-speed railway has the dual positive effect of improving economic efficiency and environmental quality.
(4) Most of the previous literature only carried out heterogeneity analysis based on the size and location of cities and did not carry out heterogeneity research on other characteristic factors of cities. In this paper, the heterogeneity analysis is carried out from the three dimensions of city size, city location, and city characteristics, which will help to more comprehensively understand the internal relationship between the opening of high-speed rail, factor allocation efficiency, and environmental pollution [14,30].The results of the heterogeneity analysis show that, from the perspective of heterogeneity at the city scale, the larger the city scale, the stronger the factor allocation efficiency optimization effect of high-speed railway opening. The environmental governance effect of opening high-speed railways is strongest for medium-sized cities, followed by large cities, and weakest for small cities. From the perspective of the heterogeneity of urban characteristics, the more abundant the financial strength and human capital of the city and the higher the level of urban administration, the stronger the environmental governance effect of the opening of a high-speed railway. The optimization effect of factor allocation efficiency and the environmental governance effect of high-speed railway openings increased in turn in the eastern, central, and western regions.

The research conclusions of this paper have important guiding significance for supporting the national improvement of high-speed railway network construction, improving the efficiency of inter-regional factor allocation, and improving the quality of urban environments. The policy implications of this paper are:

(1) Further improve the coverage of the high-speed railway network. Although the construction of high-speed railways is a difficult project with a long construction period, huge investment, and relatively long return on investment, it has a significant factor allocation efficiency optimization effect and environmental governance effect, which are particularly important for helping China accelerate the construction of a new development pattern of “dual circulation” and build a “unified domestic market.” Therefore, it is necessary to further improve the high-speed railway network to ensure that the optimization effect of factor allocation efficiency and the environmental governance effect of high-speed railway opening are fully utilized.
(2) In the future, the planning of the high-speed railway network should pay more attention to the balanced and coordinated development between regions and increase the construction of high-speed railways in the central and western regions. Since there are relatively few high-speed railway lines in the central and western regions, the marginal effect of optimizing factor allocation efficiency and environmental governance is significantly higher than that in the eastern region. Therefore, in the future, China’s high-speed railway construction should be appropriately tilted toward the central and western regions to change the incongruity of fewer high-speed railway lines and sparse high-speed railway networks in the central and western regions and vigorously promote the construction of high-speed railways in the central and western regions to accelerate the solution of the main contradiction of China’s current imbalance and insufficient development.
(3) The promotion of urban environmental governance should be “different from city to city”, and the environmental governance work of small cities or cities with relatively few financial resources, human resources, and other element endowments should focus on how to introduce high-level development factors such as capital and human capital through policy optimization rather than blindly expanding the construction of transportation infrastructure. China’s future high-speed railway construction should pay attention to further tilting towards large and medium-sized cities so as to further strengthen the radiation driving effect of large and medium-sized cities and achieve the purpose of integrated development of large, medium-sized, and small cities.
(4) Station cities along the high-speed railway line should further optimize foreign investment attraction policies and talent preferential policies, further amplify the technological innovation, foreign investment attraction, and population agglomeration effects of the high-speed railway opening, promote the free and efficient flow and distribution of multiple factors across regions, reduce the artificial policy factors that restrict the flow of factors, and realize the multi-dimensional and coordinated development between region, economy, and environment.

## Figures and Tables

**Table 1:** Descriptive statistical results.

Variable Symbol	Control Group Samples					Experimental Group Samples				
	Sample Size	Mean	Standard Deviation	Minimum	Maximum	Sample Size	Mean	Standard Deviation	Minimum	Maximum
*mis*	2806	1.838	1.992	0.036	41.122	797	1.801	1.246	0.143	8.921
*misk*	2806	1.330	0.811	0.139	10.506	797	1.239	0.550	0.441	3.948
*misl*	2806	1.228	0.514	0.128	4.941	797	1.162	0.448	0.258	2.376
*ww*	2806	65.055	84.716	0.170	912.600	797	62.394	83.680	0.070	804.680
*so*	2806	56.105	48.695	0.064	337.164	797	55.882	47.186	0.000	496.377
*HSR*	2806	0.000	0.000	0.000	0.000	797	1.000	0.000	1.000	1.000
*HSRsum*	2806	0.000	0.000	0.000	0.000	797	1.317	0.654	1.000	7.000
*open*	2806	−2.642	1.462	−8.438	2.378	797	−2.093	1.301	−6.339	1.548
*urb*	2806	3.478	0.519	2.515	4.487	797	3.683	0.505	2.515	4.487
*inf*	2806	3.263	0.581	0.445	5.875	797	3.265	0.486	1.785	5.351
*fin*	2806	0.580	0.370	−0.580	1.962	797	0.766	0.411	−0.319	2.099
*hc*	2806	4.963	8.898	0.000	80.972	797	12.411	17.413	0.000	94.699
*pop*	2806	0.094	0.247	0.000	3.806	797	0.158	0.300	0.000	2.685
*fdi*	2806	0.330	0.695	0.000	11.012	797	0.938	1.424	0.000	14.005
*cy*	2806	−0.542	0.217	−0.946	0.233	797	−0.450	0.216	−0.865	0.098
*str*	2806	−0.319	0.452	−2.362	3.279	797	−0.204	0.364	−1.358	1.414
*rgdp*	2806	9.952	0.733	7.601	12.281	797	10.670	0.488	9.089	11.810

Note: The author uses Stata to calculate and plot his table.

**Table 2:** The level of factor misallocation between prefecture-level cities in China from 2006 to 2019.

Year	Total Misallocation	Labor Misallocation	Capital Misallocation	Proportion of Labor Misallocation	Proportion of Capital Misallocation
2006	74.11%	37.72%	16.21%	50.89%	21.87%
2007	78.98%	36.69%	17.58%	46.45%	22.25%
2008	86.95%	42.53%	17.87%	48.91%	20.55%
2009	85.60%	36.01%	18.25%	42.07%	21.32%
2010	73.29%	31.32%	18.52%	42.73%	25.27%
2011	58.59%	24.69%	19.67%	42.15%	33.57%
2012	48.45%	21.15%	20.45%	43.66%	42.20%
2013	41.11%	17.97%	20.43%	43.71%	49.69%
2014	37.09%	15.65%	19.64%	42.20%	52.94%
2015	33.79%	15.16%	19.22%	44.86%	56.88%
2016	29.86%	11.39%	18.68%	38.14%	62.53%
2017	28.87%	10.98%	18.12%	38.03%	62.75%
2018	29.01%	11.18%	19.23%	38.53%	66.29%
2019	29.29%	11.83%	17.85%	40.39%	60.94%

Note: The authors calculated their results based on the inter-regional factor misallocation evaluation model constructed in this paper.

**Table 3:** The impact of high-speed rail opening on interregional factor misallocation.

Explained Variables	*Mis*	*Misk*	*Misl*	*Mis*	*Misk*	*Misl*
*hsr*	−0.3751 *** (−6.56)	−0.1304 *** (−5.74)	−0.0779 *** (−7.80)			
*hsrsum*				−0.2654 *** (−6.14)	−0.0861 *** (−4.98)	−0.0559 *** (−8.42)
Control variables	YES	YES	YES	YES	YES	YES
Entity fixed effects	YES	YES	YES	YES	YES	YES
Time fixed effects	YES	YES	YES	YES	YES	YES
Adjusted R^2^	0.6908	0.7344	0.8857	0.6919	0.7346	0.8863
Sample size	3603	3603	3603	3603	3603	3603

Note: ***, ** and * respectively indicate that they pass the significance test at the level of 1%, 5% and 10%, and the values in parentheses below coefficients are t-values.

**Table 4:** Empirical regression results of the influence mechanism of high-speed railway opening on the efficiency of factor allocation between prefecture-level cities.

Overall misallocation	**Explained variables**	***cx***	***Mis***	***Mis***	***fdi***	***Mis***	***pop***	***Mis***
	*hsr*	0.3452 *** (16.32)	−0.3751 *** (−6.56)	−0.1604 *** (2.91)	0.2477 *** (7.08)	−0.2312 *** (−4.46)	0.0211 *** (8.78)	−0.2749 *** (−5.05)
	*cx*			−0.4278 *** (−5.54)				
	*fdi*					−0.3103 *** (−4.40)		
	*pop*							−0.5311 *** (−4.19)
	Control variables	YES	YES	YES	YES	YES	YES	YES
	Time fixed effects	YES	YES	YES	YES	YES	YES	YES
	Entity fixed effects	YES	YES	YES	YES	YES	YES	YES
	Adjusted R^2^	0.8746	0.6908	0.6970	0.7844	0.6973	0.9783	0.6936
	Sample size	3603	3603	3603	3603	3603	3603	3603
Capital misallocation	**Explained variables**	***cx***	***Mis***	***Mis***	***fdi***	***Mis***	***pop***	***Mis***
	*hsr*	0.3452 *** (16.32)	−0.1304 *** (−5.74)	−0.0676 *** (−3.13)	0.2477 *** (7.08)	−0.0702 *** (−3.34)	0.0211 *** (8.78)	−0.1067 *** (−4.789)
	*cx*			−0.0865 *** (−2.59)				
	*fdi*					−0.1100 *** (−4.24)		
	*pop*							0.6364 * (1.87)
	Control variables	YES	YES	YES	YES	YES	YES	YES
	Time fixed effects	YES	YES	YES	YES	YES	YES	YES
	Entity fixed effects	YES	YES	YES	YES	YES	YES	YES
	Adjusted R^2^	0.8746	0.7344	0.7299	0.7844	0.7327	0.9783	0.7297
	Sample size	3603	3603	3603	3603	3603	3603	3603
Labor misallocation	**Explained variables**	***cx***	***Mis***	***Mis***	***fdi***	***Mis***	***pop***	***Mis***
	*hsr*	0.3452 *** (16.32)	−0.0779 *** (−7.80)	−0.0386 *** (−4.24)	0.2477 *** (7.08)	−0.0618 *** (−6.67)	0.0624 *** (7.00)	−0.0545 *** (−5.97)
	*cx*			−0.0801 *** (−8.91)				
	*fdi*					−0.0178 *** (−3.32)		
	*pop*							−0.5573 *** (−5.19)
	Control variables	YES	YES	YES	YES	YES	YES	YES
	Time fixed effects	YES	YES	YES	YES	YES	YES	YES
	Entity fixed effects	YES	YES	YES	YES	YES	YES	YES
	Adjusted R^2^	0.8746	0.9057	0.9063	0.7844	0.9041	0.9898	0.9057
	Sample size	3603	3603	3603	3603	3603	3603	3603

Note: ***, ** and * respectively indicate that they pass the significance test at the level of 1%, 5% and 10%, and the values in parentheses below coefficients are t-values.

**Table 5:** Analysis of the impact mechanism of urban factor allocation efficiency improvement on urban environmental pollution.

Industrial structure optimization effect	**Explained variables**	***str***	***so***	***so***	***ww***	***ww***
	*Mis*	−0.0188 *** (−2.75)	0.9498 ** (2.38)	0.7459 ** (1.98)	2.8680 *** (4.10)	2.5611 *** (4.16)
	*str*			−2.4219 *** (−3.91)		−2.5442 *** (4.16)
	Control variables	YES	YES	YES	YES	YES
	Time fixed effect	YES	YES	YES	YES	YES
	Entity fixed effect	YES	YES	YES	YES	YES
	Adjusted R^2^	0.8173	0.8272	0.8427	0.8455	0.8590
	Sample size	3603	3603	3603	3603	3603
Revenue-boosting effect	**Explained variables**	***agdp***	***so***	***so***	***ww***	***ww***
	*mis*	−0.5227 *** (−4.06)	0.9498 ** (2.38)	0.0291 *** (4.06)	2.8680 *** (4.10)	2.8013 *** (3.95)
	*Agdp*			−0.0001 *** (−6.64)		−1.4959 * (−1.91)
	Control variables	YES	YES	YES	YES	YES
	Time fixed effect	YES	YES	YES	YES	YES
	Entity fixed effect	YES	YES	YES	YES	YES
	Adjusted R^2^	0.8916	0.8272	0.8341	0.8455	0.8456
	Sample size	3603	3603	3603	3603	3603
Human capital agglomeration effect	**Explained variables**	***he***	***so***	***so***	***ww***	***ww***
	*Mis*	−0.4133 *** (−2.58)	0.9498 ** (2.38)	0.6605 * (1.76)	2.8680 *** (4.10)	2.7233 *** (4.10)
	*he*			−0.7240 *** (−4.01)		−0.3557 * (−1.69)
	Control variables	YES	YES	YES	YES	YES
	Time fixed effect	YES	YES	YES	YES	YES
	Entity fixed effect	YES	YES	YES	YES	YES
	Adjusted R^2^	0.9378	0.8272	0.8293	0.8455	0.8456
	Sample size	3603	3603	3603	3603	3603

Note: ***, ** and * respectively indicate that they pass the significance test at the level of 1%, 5% and 10%, and the values in parentheses below coefficients are t-values.

**Table 6:** The impact of high-speed railway opening on urban environmental pollution: analysis of the impact mechanism based on factor allocation efficiency.

Explained Variable	*mis*	*so*	*so*	*ww*	*ww*
Method	Two-Way Fixed Effects	Two-Way Fixed Effects	Two-Way Fixed Effects	Two-Way Fixed Effects	Two-Way Fixed Effects
*hsr*	−0.3751 *** (−6.56)	−6.0268 *** (−4.44)	−5.7816 *** (−4.29)	−9.1228 *** (−3.97)	−8.2972 *** (−3.63)
*Mis*			0.7207 * (−1.76)		2.6797 *** (4.04)
Control variables	YES	YES	YES	YES	YES
Time fixed effect	YES	YES	YES	YES	YES
Entity fixed effect	YES	YES	YES	YES	YES
Adjusted R^2^	0.6908	0.8296	0.8298	0.8452	0.8258
Sample size	3603	3603	3603	3603	3603

Note: ***, ** and * respectively indicate that they pass the significance test at the level of 1%, 5% and 10%, and the values in parentheses below coefficients are t-values.

**Table 7:** Analysis of heterogeneity of city scale: the impact of high-speed railway opening on the factor allocation efficiency of cities of different scales.

Explained Variable	*Mis*		
City Scale	Large Cities	Medium-Sized Cities	Small Cities
*hsr*	−0.3035 *** (−3.40)	−0.1401 ** (−2.04)	−0.0754 (−0.75)
Control variables	YES	YES	YES
Time fixed effect	YES	YES	YES
Entity fixed effect	YES	YES	YES
Adjusted R^2^	0.7607	0.7324	0.6372
Sample size	1083	1047	1435

Note: ***, ** and * respectively indicate that they pass the significance test at the level of 1%, 5% and 10%, and the values in parentheses below coefficients are t-values.

**Table 8:** Analysis of heterogeneity of city scale: the impact of high-speed railway opening on environmental pollution in cities of different scales.

Explained Variable	*so*			*ww*		
City Scale	Small Cities	Medium-Sized Cities	Large Cities	Small Cities	Medium-Sized Cities	Large Cities
*hsr*	−3.5107 * (−1.74)	−4.9972 ** (−2.29)	−4.8459 * (−1.74)	−2.5278 (−0.55)	−18.4636 *** (−3.39)	−5.9752 * (−1.93)
Control variable	YES	YES	YES	YES	YES	YES
Time fixed effect	YES	YES	YES	YES	YES	YES
Entity fixed effect	YES	YES	YES	YES	YES	YES
Adjusted R^2^	0.8397	0.7662	0.8405	0.6073	0.8281	0.9246
Sample size	1435	1047	1083	1435	1047	1083

Note: ***, ** and * respectively indicate that they pass the significance test at the level of 1%, 5% and 10%, and the values in parentheses below coefficients are t-values.

**Table 9:** Heterogeneity analysis of urban characteristics of environmental governance effects of high-speed railway opening.

**Explained variable**	***ww***	***so***	***ww***	***so***
**Sample classification**	**Cities with high fiscal pressure**		**Cities with low fiscal pressure**	
*hsr*	−7.6307 ***	−1.0233	−12.0461 ***	−7.9263 ***
	(−2.65)	(−0.58)	(−3.32)	(−3.84)
Adjusted R^2^	0.6523	0.8315	0.858	0.8217
Sample size	1798	1798	1792	1792
**Explained variable**	***ww***	***so***	***ww***	***so***
**Sample classification**	**Cities with high human capital**		**Cities with low human capital**	
*hsr*	−0.0508 **	−5.4373 ***	0.0091	−0.3819
	(−1.96)	(−3.68)	(−0.09)	(−0.10)
Adjusted R^2^	0.8785	0.8231	0.7946	0.8792
Sample size	2691	2692	879	879
**Explained variable**	***ww***	***so***	***ww***	***so***
**Sample classification**	**Provincial capitals**		**Non-provincial capitals**	
*hsr*	−16.4027 ***	−14.8722 ***	−8.1165 ***	−4.0355 ***
	(−3.27)	(−3.24)	(−3.17)	(−2.73)
Adjusted R^2^	0.9553	0.7123	0.8072	0.8339
Sample size	326	326	3277	3277

Note: ***, ** and * respectively indicate that they pass the significance test at the level of 1%, 5% and 10%, and the values in parentheses below coefficients are t-values.

**Table 10:** Analysis of heterogeneity in urban areas: the impact of high-speed railway opening on the efficiency of urban factor allocation in different regions.

Explained Variable	*Mis*			*Misk*			*Misl*		
City Scale	Eastern Region	Central Region	Western Region	Eastern Region	Central Region	Western Region	Eastern Region	Central Region	Western Region
*hsr*	−0.0629 (−1.12)	−0.2619 ** (−2.31)	−0.6595 *** (−5.09)	−0.0104 (−0.40)	−0.1634 * (−2.08)	−0.3015 *** (−5.71)	−0.0163 (−1.36)	−0.0559 *** (−3.69)	−0.0940 *** (−4.59)
Control variables	YES	YES	YES	YES	YES	YES	YES	YES	YES
Time fixed effect	YES	YES	YES	YES	YES	YES	YES	YES	YES
Entity fixed effect	YES	YES	YES	YES	YES	YES	YES	YES	YES
Adjusted R^2^	0.8651	0.6428	0.7062	0.8445	0.7213	0.7512	0.9641	0.8131	0.8551
Sample size	1248	1313	1042	1248	1313	1042	1248	1313	1042

Note: ***, ** and * respectively indicate that they pass the significance test at the level of 1%, 5% and 10%, and the values in parentheses below coefficients are t-values.

**Table 11:** Analysis of heterogeneity in urban areas: the impact of high-speed railway opening on urban environmental pollution in different regions.

Explained Variable	*so*			*ww*		
City Scale	Eastern Region	Central Region	Western Region	Eastern Region	Central Region	Western Region
*hsr*	−0.0730 (−0.04)	−5.2151 ** (−2.21)	−15.0155 *** (−4.29)	0.0631 (0.03)	−12.1843 *** (−2.70)	−23.6066 *** (−3.83)
Control variables	YES	YES	YES	YES	YES	YES
Time fixed effect	YES	YES	YES	YES	YES	YES
Entity fixed effect	YES	YES	YES	YES	YES	YES
Adjusted R^2^	0.8509	0.8162	0.8061	0.8656	0.7751	0.6281
Sample size	1248	1313	1042	1248	1313	1042

Note: ***, ** and * respectively indicate that they pass the significance test at the level of 1%, 5% and 10%, and the values in parentheses below coefficients are t-values.

## Data Availability

The data presented in this study are available on request from the corresponding author.
